# Conscientious objection to abortion in the developing world: The correspondence argument

**DOI:** 10.1111/dewb.12302

**Published:** 2020-12-04

**Authors:** Himani Bhakuni, Lucas Miotto

**Keywords:** conscientious objection, abortion, global south, nurses and midwives, correspondence thesis

## Abstract

In this paper we extend Heidi Hurd’s “correspondence thesis” to the termination of pregnancy debate and argue that the same reasons that determine the permissibility of abortion also determine the justifiability of acts involving conscientious objection against its performance. Essentially, when abortion is morally justified, acts that prevent or obstruct it are morally unjustified. Therefore, despite conscientious objection being legally permitted in some global south countries, we argue that such permission to conscientiously object would be morally wrong in cases of morally justifiable termination of pregnancy. After presenting and defending our “correspondence argument” we suggest that conscientious objection should be denied as a matter of public health policy in developing counties, even in cases where adequate referral services are possible. Towards the end, we extend our argument to midwives, nurses, and prospective students in the field. Given their essential position in resource‐poor contexts; they too have no claim to conscientious objection.

## 1 INTRODUCTION

The reasons that morally justify an act also determine whether third parties are morally justified in permitting or preventing that same act. This is what Heidi Hurd calls the “*correspondence thesis*” in her book *Moral Combat*.^1^ This paper argues that the correspondence thesis can help us settle the debate around the permissibility of conscientious objection to termination of pregnancy. By developing what we call “the correspondence argument”, we argue that legal provisions that allow for conscientious objection to morally justified termination of pregnancy are morally wrong. Taking stock of the health care realities in the global south we argue that, as a matter of public policy, developing countries should leave no room for conscientious objection to morally justified termination of pregnancy.

Section 2 outlines the correspondence argument. Section 3 addresses how the correspondence argument fares in the global south and argues for the denial of conscientious objection even in cases where referral services are possible. Section 4 discusses a possible objection based on the moral integrity of doctors. Section 5 clarifies the scope of our main argument and extends it to midwives, nurses, and students of medicine and nursing who have an important role to play in providing abortion services in the global south.

## 2 THE CORRESPONDENCE ARGUMENT

Consider the following pair of cases:

(Charity) Pooja donates 10% of her monthly income to a legitimate non‐profit organisation working on Malaria aid. Pooja does not need the money and her donation directly brings relief to hundreds of people every month. Samir, her husband, believes that Pooja is wasting her money and tries to stop Pooja from donating to the organisation.

(Shell Charity) Pooja donates 10% of her monthly income to a fraudulent organisation which claims to be working on Malaria aid. Pooja does not need the money, but unbeknownst to her the amount donated is used to finance human trafficking and terrorist groups. Samir, her husband, believes that Pooja is wasting her money and tries to stop Pooja from donating to the organisation.

These two cases differ in various respects. One important difference for our purposes concerns the moral status of Samir’s actions. Samir’s attempt to stop Pooja from donating in (Charity) is morally condemnable. Pooja is morally permitted to donate her surplus income to good causes and to directly help those in need. In fact, some would even argue that she has a moral duty to do so.^2^ But the same is not true in (Shell Charity). In this case, Pooja is in the wrong and Samir is morally permitted, if not required, to prevent Pooja from donating to the organisation. What these two cases suggest is that whether Samir is morally permitted (or required) to prevent Pooja’s donation or not depends on the moral status of Pooja’s donation. Samir is morally permitted from preventing Pooja’s wrongful donation; but he is not permitted to prevent Pooja’s rightful donation.

This much is consistent with common sense morality. It is also the core of what Heidi Hurd has baptised as the “*correspondence thesis*”. The correspondence thesis concerns the justification of co‐dependent actions. It asserts that “the justifiability of an action determines the justifiability of permitting or preventing that action.”^3^ The underlying assumption is that an action cannot be at the same time right and wrong^4^; so, if an agent is, on balance, justified in performing an act, another agent cannot be simultaneously, on balance, justified in preventing that same act. Similarly, if one’s action is, on balance, wrong, someone else cannot be, on balance, justified in permitting that same act. In a nutshell: if an action is wrong, permitting it is also wrong and if an action is right, preventing it is wrong.

The correspondence thesis has interesting implications for the discussions on conscientious objection to termination of pregnancy. In general terms, this thesis can be used in an argument against the practice of conscientiously objecting to termination of pregnancy. The argument – we call it “the correspondence argument” – runs as follows:

*The correspondence argument*

1. Preventing, obstructing, or adding a burden on morally justified termination of pregnancy is morally wrong.
2. Conscientiously objecting to termination of pregnancy often prevents, obstructs, or adds burdens on morally justified termination of pregnancy.
3. Therefore, conscientiously objecting to morally justified termination of pregnancy is *ceteris paribus* morally wrong.

Let us clarify a few things about this argument. First, the argument centres on conscientious objection to morally justified termination of pregnancy; cases where women have the “right to abortion”, as it were. Our discussion, therefore, will focus on cases where individuals conscientiously object to morally justified abortion laws and policies. This focus, however, might lead some to think that our correspondence argument begs the question against those who conscientiously object to abortion laws and policies precisely because they believe that such laws and policies are morally wrong. However, “[an] argument [for conscientious objection] *must* proceed on the assumption that the law is morally valid.”^5^ That is because the discussion on the permissibility of conscientious objection to morally unjust laws and policies would most likely be trivial and uninteresting. After all, in such circumstances not only would one be clearly permitted to conscientiously object, but a case could also be made for stronger, and perhaps more apt, responses against such laws and policies (e.g., civil disobedience and reform). In other words, the philosophical and practical interest in conscientious objection and the challenges associated with its justification lies in “showing that a person is entitled not to do what it would otherwise be his moral duty to do simply because he *wrongly* believes that it is wrong for him to do so.”^6^

Hence, by focusing on cases where abortion is morally justified, we are not assuming something that the defenders of conscientious objection dispute. Quite the opposite. We are assuming something that is needed to render the debate around the justification of conscientious objection interesting and relevant.

Despite defenders of conscientious objection sharing the assumption that abortion is morally justified (if only for the sake of discussion) and despite there being independent reasons to believe that the typical abortion both in the developing and the developed world is indeed morally justified,^7^ there are circumstances where the correspondence argument does not apply in a straightforward manner. These are circumstances where the moral status of an abortion or an abortion‐related policy is unclear or contested. We will briefly discuss such cases later in the paper.

The second clarificatory point about our argument is that we are not assuming that conscientious objection to abortion is intrinsically wrong. Our point is that conscientious objection to abortion is *ceteris paribus* wrong; it is wrong only in the circumstances where it prevents, obstructs, or adds burdens on termination of pregnancy. The role of the *ceteris paribus condition* is to accommodate conscientious objection in circumstances where it does not prevent, obstruct or adds burdens on morally justified abortion. We will say a few more words about this point later while discussing some practical concerns about conscientious objection in the global south.

Third, and relatedly, the first premise of the correspondence argument states that it would also be wrongful to *obstruct* and to *add a burden* 8 on morally justified termination of pregnancy—and not just to prevent it. Hence, one may worry that this premise does not directly follow from the correspondence thesis. This is true: despite being based on the correspondence thesis, the first premise has a slightly broader scope than the referred thesis. True as it may be, this is hardly a problem for the first premise’s overall plausibility. That is because the same reasons and intuitions that warrant the correspondence thesis would warrant a similar, though slightly broader, thesis. If an action is, on balance, morally justified, *interferences* with that action are morally wrong. On the assumption that an action cannot be right and wrong at the same time, it would be impossible for an agent to be, on balance, morally justified in performing an action while another agent is simultaneously, on balance, justified in interfering with that action’s performance (by obstructing or adding burdens on it).^9^

Having presented our correspondence argument with some clarifications, let us examine how this argument would fare in the global south.

## 3 CONSCIENTIOUS OBJECTION IN THE GLOBAL SOUTH

In most countries in the global south legal termination of pregnancy is limited to situations where the continued pregnancy would pose a danger to the woman’s life or health. In a few countries abortion is legal on health or socio‐economic grounds, and in others abortion services are available on request.^10^ However, access to abortion services in these countries is oftentimes constrained by doctors and nurses who refuse to perform or assist in the procedure on the basis of religious beliefs or other conscience‐based convictions. This is primarily because most countries that legally allow abortion, whether in restricted cases or otherwise, also have legal provisions for conscientious objection.^11^ But if the correspondence argument is right, those legal provisions that allow for conscientious objection wrong women who have a morally justified claim to terminating their pregnancy.

The correspondence argument holds that it would be wrongful to *prevent*, *obstruct*, and to *add burdens* on women seeking morally justified termination of pregnancy. While most commentators would agree that conscientious objection is wrong when it *prevents* access to abortion services, they would still insist that conscientious objection should be allowed whenever adequate referral services are available.^12^ Let us place this within the global south context. Over 45% of World Health Organisation (WHO) member states report to have less than 1 physician per 1000 population.^13^ Some countries in the global south have as little as 7 physicians per 10,000 population.^14^ Access to adequate health‐care services in the global south differ amongst urban and rural settings with most health‐care providers opting to cater to the urban populace.^15^ WHO’s data serves well to show that if even one conscientious objector refuses to perform abortion it would detrimentally impact access to legally and morally permitted abortion services for a significant number of women, especially burdening women from rural areas who have to travel far to find abortion providers. Given the ground realities of health systems in most developing countries, conscientious objection would, in the majority of cases, pose serious burdens on women and, in some cases, effectively amount to *prevention* of morally justified termination of pregnancy.

Other reasons point in the same direction. Recent qualitative studies from developing countries suggest that abortion providers often assert conscientious objection as a means to oppose abortion on wide‐ranging grounds which negatively impacts access and increases the incidence of unsafe abortions.^16^ A study from South Africa found that “a range of hospital or clinic staff, even those not directly involved in abortion provision, refused or provided unnecessary barriers to those providers who wanted to provide [abortion related] care”.^17^ These studies suggest that, in practice, conscientious objection is often abused, poses serious *burdens* on women, and is overall detrimental to morally and legally justified right to abortion.

This brings us to considerations about health policy in developing countries. First, in most developing countries the doctor‐patient relationship is hierarchical, trust‐based, and asymmetrical,^18^ especially considering the limited access to health care services for huge swaths of population. Within this context, abortion providers who exercise conscientious objection are effectively employing their position of authority and trust to enforce their personal beliefs on women who are utterly dependent on them for essential healthcare. Second, referrals made by conscientious objectors in developing countries with dominant public health care systems could potentially expose women to multiple layers of bureaucracy. In such context, referrals are doubly risky; it not only increases the risk of physical and mental health issues associated with delay, but it also risks further demoralising vulnerable sectors of the population. We should not assume that conscientious objection is acceptable *just because* there is a working referral system in place. We must balance the benefits of such referrals with the burdens they place on women seeking abortion.

It would not be far‐fetched to assume that many women seeking termination of pregnancy in developing countries might have encountered several hardships *before* seeking a health‐care provider for such termination. Domestic abuse, rape, and financial constraints are obvious, and unfortunately endemic, examples. But specially in places where abortion is culturally condemned, women seeking termination are often exposed to more subtle, though serious, forms of hardship: social ostracism, family pressure, emotional abuse, and decision anxiety are some examples that come to mind. To allow such women to be denied their morally justified claim to termination by their doctors and to be asked to withstand yet another hurdle (through referral) borders cruelty and is doubtlessly demoralising.

Conscientious objection in developing countries often *prevents* morally and legally justified abortion and in almost all cases *adds burdens* on women seeking termination. As a matter of principle, the state must ensure that its policies do not leave vulnerable groups worse off. Public policy ought not to work as a *de facto* deterrent to morally justified abortion or as a tool to demoralise women seeking to satisfy their morally justified rights. Public policies that affect an entire peoples’ rights and health must not look at cases in isolation, especially when all other national policies are made with the typical case in mind. There can be detailed provisions for the *very* exceptional cases of conscientious objection where no burdens are added on women seeking abortion. But as a matter of general policy, governments of developing countries have good reasons to *deny* conscientious objection, even when adequate referrals are sometimes available. Some, however, may object to this policy proposal on the basis of considerations about doctors’ integrity and democratic values. The next section addresses such objection.

## 4 INTEGRITY OF THE CONSCIENTIOUS OBJECTOR WITHIN CONTEXT

Objectors to our argument might argue that it would be wrong to deny the means to preserve the moral integrity of those who have strong moral or personal convictions against abortion. A democratic state should respect a plurality of views and opinions and should, as far as possible, not require individuals to give up moral convictions that might be central to their self‐conception or identity.^19^ There are at least a couple of reasons to resist this objection.

The first is that a conditional provision that permits conscientious objection *only* when adequate referral services are possible does not seem to properly overcome the integrity problem. That is because, as pointed out by some, referring to do wrong is still wrong.^20^ One does not seem to preserve one’s moral integrity by saying “I don’t abort and abortion is wrong, but here is the contact of someone who does”. In fact, this sort of behaviour strikes us as deeply hypocritical. Conscientious objection is an important tool for excusing oneself from partaking in practices one morally condemns. Though it might be important to be given a chance to preserve one’s conscience, conscientiously objecting *only* to keep your own hands clean and to enforce a private sense of justice on others should be condemned.^21^ Such exercise of conscience should not come at the expense of putting fundamental, and morally justified, rights of women at risk. Contrary to military conscription, where individuals use conscientious objection to oppose being required to join the army against their will, no one is “plucked at random and required by law to deliver abortion”.^22^ When one’s conscience clashes with the professional duties of a doctor, one should be conscious of becoming a doctor.

The second reason to resist the integrity‐based objection is that when abortion is morally justified, *anyone* who believes it to be wrong is mistaken. If they are mistaken, they have no reasonable complaints to refrain from performing it. We must be wary of allowing public health policies to be guided by unreasonable complaints. No doubt that in ideal circumstances it would be desirable for a democratic state to accommodate everyone’s personal and moral beliefs, even if these beliefs are ultimately mistaken. But in the real world, and specially in the developing world, such accommodation comes at the expense of amplifying the burdens of vulnerable groups. That mistaken moral or personal views will be violated if we choose to deny doctors to conscientiously object to morally and legally justified termination of pregnancy doesn’t strike us as a dilemma, but as a good bargain.

We must stress that we are aware of an epistemic barrier that affects our Correspondence Argument: sometimes we do not know which instances of abortion are morally justified and which are not. Some might argue that because there is uncertainty with regards to the moral status of particular instances of abortion, we might want to adopt a more tolerant position on conscientious objection. However, we should not be hasty in embracing tolerance and in giving more room to conscientious objection. The consequences of conscientious objection are detrimental to women. But the consequences of not allowing one to conscientiously object to a particular abortion are less clear. The compromise in such situations would be to not allow blanket conscientious objection, but permit some leeway for internal policies in well‐staffed hospitals. This leeway would be in line with the *ceteris paribus* condition in our Correspondence Argument. But for this condition to obtain, referrals must be entirely administrative and the women seeking abortion must not be informed of such referrals (to avoid mental anguish) and no other burden to access must be placed in their way to exercise their right to abortion. As this exception can well be incorporated into our policy proposal of *denying* conscientious objection, we see no good reasons to surrender to the integrity challenge and to allow blanket conscientious objection in the global south.

In the next section we conclude by further clarifying the scope of our Correspondence Argument and asserting that the denial of conscientious objection should be extended to all current and future abortion service and care providers.

## 5 CONCLUSION: DOCTORS, STUDENTS, AND MIDWIVES

We have argued that conscientious objection to morally justified termination of pregnancy should have no place in the developing world. There are, however, no good reasons to *restrict* the conclusion of our correspondence argument to the developing world. Even though the burdens that conscientious objection places on women seeking abortion are more prominent and frequent in the developing world due to the systematic inadequacy of healthcare resources, it is not altogether inconceivable that conscientious objection might sometimes pose the same burdens on women seeking abortion in developed countries. In fact, in places like Italy, for example, conscientious objection notoriously burdens women seeking termination despite the existence of legal regulations limiting its exercise.^23^ Preventing, obstructing, and burdening morally justified termination of pregnancy is as morally wrong in the developing world as it is in the developed world. Thus, as per the correspondence argument, conscientious objection should be ruled out whenever and wherever its exercise prevents, obstructs or adds burdens on women seeking morally and legally justified termination of pregnancy.

Like most writers on the topic, we seem to have focused on *doctors*. As a matter of clarification, we must add that the correspondence argument need not—and should not—be restricted to *doctors*; it applies to all and sundry. According to our correspondence argument, it would not only be wrong for *doctors* to conscientiously object to morally justified termination of pregnancy, but also to midwives, nurses, and prospective students and trainees of medicine, nursing, and midwifery.

A recent study called out the invisibility of midwives and nurses in the debate concerning conscientious objection.^24^ This is surprising since the WHO recognises midwives and nurses as the most essential sexual and reproductive healthcare providers in developing countries.^25^ Though our argument extends to midwives, we are also aware of the unregistered (hence, unregulated) midwives that abound in some rural areas in the global south. The social costs and the overall viability of depriving registered and unregistered midwives from conscientiously objecting to termination are still unclear to us. For brevity’s sake we cannot discuss these here. But we hope that our argument invites further reflection and empirical investigation on the matter.

## CONFLICT OF INTERESTS

None.

## Footnotes

^1^
Hurd, H. (1999). Moral Combat: The Dilemma of Legal Perspectivalism. Cambridge: Cambridge University Press.

^2^
Singer, P. (2010). The life you can save: How to do your part to end world poverty. New York: Penguin Random House.

^3^
Hurd, op. cit. note 1: 3.

^4^
In this paper we (loosely) assume that “right”, “morally justified”, and “morally permitted” are synonymous. We also assume that “wrong”, “morally unjustified”, and “morally prohibited” are synonymous.

^5^
Raz, J. (1979). The authority of law: Essays on law and morality. Oxford: Oxford University Press. Our emphasis. Note that “morally valid” means more than just procedural adequacy. That is, a law that is in line with a country’s constitutional provisions and legislative procedures can still be immoral. Here we (and most likely Raz) understand moral validity in a robust sense rather than a purely procedural sense. We extend our argument to cases where the moral status of abortion is contested or opaque later in the paper.

^6^
Ibid.: 277 Our emphasis.

^7^
Most of the controversies surrounding the morality of abortion revolve around late abortions. With the exception of religion‐based objectors, it is largely agreed that early termination (those taking place in the first trimester) is morally permitted. See Greasley, K., & Kaczor, C. (2017). Abortion Rights: For and Against. Cambridge: Cambridge University Press. As it happens, the vast majority of abortions around the world (in both developing and developed countries) take place in the early stages of pregnancy. (For a comparison of the proportion of first trimester abortions with second trimesters, see Boland, R. (2010). Second trimester abortion laws globally: actuality, trends and recommendations. *Reproductive Health Matters*. *18*(36), 67–89. 10.1016/S0968-8080(10)36521-9; Dalvie, S.S. (2008). Second Trimester Abortions in India. *Reproductive Health Matters*. *16*(sup31), 37–45. 10.1016/S0968-8080(10)36521-9) Moreover, Legislatures in all democratic countries are sensitive to these moral concerns and legislations on abortion are usually preceded by extensive legislative debate.

^8^
Note that we may distinguish between different kinds of burdens. Some burdens are trivial, others are necessary for the performance of an action, and others unnecessarily raise the costs of performing an action. For example, to get an abortion a woman must make an appointment with a doctor, in a trivial sense this could be interpreted as a burden. Relatedly, to perform an abortion a woman would also have to give informed consent, which would involve a consent counselling/information session and signing of papers and consequently a delay in performance of abortion. This also could be interpreted as a burden, but these burdens are not the kind of burdens that would have similar (or same) moral weight as prevention or obstruction. For our argument we are interested only in those kinds of burdens that pose unnecessary costs.

^9^
Of course, if an agent only has a Hohfeldian liberty (or privilege) to perform an action, then it would indeed be possible for others to be free to interfere with the action. But Hohfeldian liberties are weak and “may not be worth very much to us if [they are] not protected by certain claim‐rights”. Perry, T.D. (1977). A Paradigm of Philosophy: Hohfeld on Legal Rights. *American Philosophical Quarterly*. *14*(1), 41–50. The cases we are concerned with are cases where women have the so‐called “right to abortion”, meaning that they are both free to terminate pregnancy and have a claim that others allow them or facilitate the procedure. Practically speaking, it would be pointless for women to fight for the “right to abortion” if such “right” was understood as a simple Hohfeldian liberty. For a more detailed defence of the correspondence thesis against objections based on Hohfeldian liberties, see Hurd, op. cit. note 1: 280–284.

^10^
Centre for Reproductive Rights. (2020). World’s Abortion Laws. Retrieved June 24, 2020, from https://reproductiverights.org/worldabortionlaws

^11^
World Health Organisation. (2020). Global Abortion Policies Database. Retrieved June 24, 2020, from https://abortion‐policies.srhr.org/

^12^
Wicclair, M.R. (2011). Conscientious Objection in Health Care: An Ethical Analysis. Cambridge: Cambridge University Press; Brock, D.W. (2008). Conscientious refusal by physicians and pharmacists: who is obligated to do what, and why? *Theoretical Medicine and Bioethics*. *29*(3), 187–200; LaFollette, E., & LaFollette, H. (2007). Private conscience, public acts. *Journal of Medical Ethics*. *33*(5), 249–254; Savulescu, J. (2006). Conscientious objection in medicine. *BMJ*. *332*(7536), 294–297.

^13^
World Health Organisation. Global Health Observatory (GHO) data. (2019). Retrieved June 24, 2020, from http://www.who.int/gho/health_workforce/physicians_density/en/.

^14^
Ibid.

^15^
Strasser, R., Kam, S.M., & Regalado, S.M. (2016). Rural Health Care Access and Policy in Developing Countries. *Annual Review of Public Health*. *37*, 395–412.10.1146/annurev-publhealth-032315-02150726735432

^16^
Freeman, E., & Coast, E. (2019). Conscientious objection to abortion: Zambian healthcare practitioners’ beliefs and practices. *Social Science & Medicine*. *221*, 106–114; Montero, A., & Villarroel, R. (2018). A critical review of conscientious objection and decriminalisation of abortion in Chile. *Journal of Medical Ethics*. *44*(4), 279–283; Coppola, F., Briozzo, L., Nozar, F., Fiol, V., & Greif, D. (2016). Conscientious objection as a barrier for implementing voluntary termination of pregnancy in Uruguay: Gynecologists’ attitudes and behavior. *International Journal of Gynecology & Obstetrics*. *134*, S16–S19; Harries, J., Cooper, D., Strebel, A., & Colvin, C.J. (2014). Conscientious objection and its impact on abortion service provision in South Africa: a qualitative study. *Reproductive Health*. *11*(1), 16.

^17^
Harries, Cooper, Strebel, & Colvin, op. cit. note 16: 16.

^18^
Subramani, S. (2018). The moral significance of capturing micro‐inequities in hospital settings. *Social Science & Medicine*. *209*, 136–144; Gopichandran, V., & Chetlapalli, S.K. (2013). Dimensions and Determinants of Trust in Health Care in Resource Poor Settings – A Qualitative Exploration. *PLoS ONE*. *8*(7), e69170; Rees, C.E., Knight, L.V., & Wilkinson, C.E. (2007). Doctors being up there and we being down here: A metaphorical analysis of talk about student/doctor–patient relationships. *Social Science & Medicine*. *65*(4), 725–737.10.1016/j.socscimed.2007.03.04417493722

^19^
For similar arguments, see Brock, op. cit. note 12: 187–200; Benn, P. (2007). Conscience and Health Care Ethics. In R. Ashcroft, A. Dawson, H. Draper, & J. MacMillan (Eds.), Principles of Health Care Ethics (pp. 345–350). John Wiley & Sons, Ltd.

^20^
Card, R.F. (2014). Reasonability and Conscientious Objection in Medicine: A Reply to Marsh and an Elaboration of the Reason‐Giving Requirement. *Bioethics*. *28*(6), 320–326; Kelleher, J.P. (2010). Emergency Contraception and Conscientious Objection. *Journal of Applied Philosophy*. *27*(3), 290–304.10.1111/bioe.1202223796253

^21^
We would like to thank an anonymous reviewer for pressing us on this point.

^22^
Greasley, K. (2017). Arguments About Abortion: Personhood, Morality, and Law. Oxford University Press.

^23^
According to expert reports, up to 75% of gynaecologists conscientiously object to abortion in Italy. Some commentators have discussed the burdens posed by conscientious objection in the Italian context. Evidence shows that this high incidence is responsible for delays, unsafe abortions, and financial burdens. For discussion, see Autorino, T., Mattioli, F., & Mencarini, L. (2020). The impact of gynecologists’ conscientious objection on abortion access. *Social Science Research*. *87*. See also, International Planned Parenthood Federation – European Network (IPPF‐EN) v. Italy, Complaint No. 87/2012, Eur. Comm. Soc. R., paras. 82‐85 (2014).

^24^
Fleming, V., Frith, L., Luyben, A., & Ramsayer, B. (2018). Conscientious objection to participation in abortion by midwives and nurses: a systematic review of reasons. *BMC Medical Ethics*. *19*(1), 31.10.1186/s12910-018-0268-3PMC592318829703258

^25^
World Health Organisation. (2011). The Role of Primary Health‐Care Providers in Sexual and Reproductive Health Results from an Intercountry Survey. Retrieved June 24, 2020, from https://www.who.int/reproductivehealth/publications/health_systems/PHC_Supplement1.pdf

